# Carrying of Wounded in the Trenches

**Published:** 1918-10

**Authors:** 


**The Carrying of Wounded in Trenches.** By Lieut. B. GRAVES. R. A. M. C., *Journal of Royal Army Medical Corps*, April, 1918. Vol. XXX, No. 4. page 432.

Compactness is the first essential for the transportation of wounded from a fire trench. In the majority of cases no harm will result to an injured man by flexion of the legs, provided that his body is maintained in a position of reasonable comfort without any auxiliary effort on his part.

The task of the stretcher bearer is one which is exceedingly tiring and arduous, and any device which will lessen the difficulty will greatly increase the efficiency with which the task is carried out.

In sections of trenches where the ordinary stretcher cannot possibly be utilized, resort has been made to a rubber ground sheet upon which the patient has been dragged until the difficult part has been covered. An improvement upon this is a sheet of canvas with a rope ring at each of its corners. It is a good plan to place such a sheet upon the stretcher before the placing of the patient, thus facilitating his removal if, and when, it becomes necessary.

The vertical position, of course, occupies the least space. The difficulty connected with this is that, while the patient's feet must of necessity clear the ground, his head is often unduly exposed to fire. A similar objection pertains to the hammock carried shoulder-high, and this, moreover, involves a great strain on the part of the stretcher bearers, particularly in counteracting a tendency to stumble. Furthermore, the *upward pushing* effort required to ease the weight from the shoulders, from time to time, is more fatiguing than the *upward pull* of the handles of a low-slung apparatus.

The author has devised a trench carrier which has the following advantages over the old pattern :

1. It does not require the assistance of an extra man, or extra men, to mount the patient. This avoids the discomfort to the patient of superfluous manipulation and the necessity of conscious effort on his part to maintain the position.

2. It lessens the labor of the stretcher bearers.

3. The carrier is at no point wider than the patient.

4. The direction of the weight of transport is *directly downwards*.

The apparatus consists of a canvas sling, hung with a semi-circular dip, attached to a steel tube, the straight ends of which are so jointed that they can drop through a quarter of a circle to the vertical position, while that portion passing over the sling takes the form of a semi-circle, describing thus with the sling an approximate circle. The sling is attached to one end by means of a horizontal curved cross-bar, and to the other end by a hook. (The length of the canvas must be such that the centre of gravity of the patient will fall below the horizontal level of the handles.) At this point a short cross-bar with upturned ends is fixed, with a similar one placed a little beyond the joint of the straight end. These cross-bars serve as supports for the patient's legs. At the base of the semi-circular bar spanning the sling are three hooks, to any of which the extremity of the sling can be attached if a change of position be desired.

When the bearers come to a corner, or wish to pause for a rest, they drop the jointed extremities and hold by the short length which remains. At many of the less acute corners, it is only necessary to lower one of the ends. The advantage of this single-handled carrier is that the disposition thereof is not affected by the sudden removal of one hand; incidentally, it does not twist laterally to unsafe angles when this occurs, as would the old two-handled plane surface carrier.

The downward weight of support is borne by a strap passed round the opposite shoulder — an obvious advantage compared to the wasted effort caused by the strain on the back of the neck by the ordinary stretcher sling.

From the medical point of view, it may be stated that flexion is by no means uncomfortable for the patient, provided he is *secure*, and is not required to make any effort to remain so.

An improved pattern of the ordinary stretcher for use in communication trenches could be made by diminishing the width of one end, and substituting the two handles at that end by a single central one; with this it would be necessary to tie a bandage or puttee around the patient's feet. This alteration would materially assist negotiation of curves.

A jointed toboggan on three rollers, drawn by a short length of rope, is a suggested device for removing patients from No Man's Land without the necessity of stretcher bearers standing bolt upright.

The writer has also used a device for the recumbent patient somewhat in imitation of the steel tube carrier, made of wood. It is suggested that if improved by diminishing the width of the front end, and substituting a single hinged central handle as suggested above for the ordinary carrier, it would serve a good purpose in spite of the fact that a few corners might be found round which it would not pass

Finally, no one device can be capable of meeting every contingency, but the writer considers that this pattern seems to cover more than any other he has tried.

